# Surveillance study of asymptomatic and presymptomatic coronavirus disease 2019 (COVID-19) in care homes in Northern Ireland

**DOI:** 10.1017/ice.2020.1284

**Published:** 2020-10-20

**Authors:** Claire Neill, Muhammad Sartaj, Lorna Holcroft, Syed Shahzad Hasan, Barbara R. Conway, Mamoon A. Aldeyab

**Affiliations:** 1 Public Health Agency, Belfast, Northern Ireland; 2 Department of Pharmacy, School of Applied Sciences, University of Huddersfield, Huddersfield, United Kingdom

*To the Editor—*Recent studies have highlighted the potential for transmission of severe acute respiratory syndrome coronavirus 2 (SARS-CoV-2) from individuals who are symptomatic, presymptomatic, and asymptomatic of infection.^1–3^ This is of particular concern in high-risk settings, such as long-term care facilities. Care-home residents may be more vulnerable to infection with an increased likelihood of risk factors, including age and pre-existing comorbidities.^4,5^ Differentiating asymptomatic from presymptomatic infection and the range of possible symptoms associated with coronavirus disease 2019 (COVID-19) in different populations remains of interest. The potential for an atypical presentation of COVID-19 in care-home residents has been reported.^6^ This study was conducted to identify the rate of asymptomatic and presymptomatic COVID-19 in care-home residents and staff, within settings where recent cases of COVID-19 had been identified, including potential atypical presentations of those testing positive for the virus.

This is a report on the results of an outbreak investigation that was conducted as part of public health practice to manage the outbreak, to support the wider public health surveillance, and to inform policy decisions regarding SARS-CoV-2 testing in care homes. As such, the work did not require Research Ethics Committee approval, which is in keeping with the UK Health Research Authority’s guidance.

This prospective study with a follow-up review on day 7 was carried out in 5 care homes reporting recent outbreaks of COVID-19 to the Public Health Agency in Northern Ireland. If an individual was symptomatic at the time of sampling, or if they had been symptomatic within the 14 days prior to the test, it was recorded. These data included typical symptoms of cough, fever, or shortness of breath, as well as any atypical symptoms: sore throat, sneezing, nasal discharge/congestion, wheeze, hoarseness, chest pain, acute deterioration, malaise, nausea, confusion, dizziness, diarrhoea, myalgia, headache, chills, or anosmia. A follow-up review was conducted with the care homes 7 days after testing. We conducted this assessment to determine whether anyone who tested positive for SARS-CoV-2 while asymptomatic subsequently developed any symptoms during this 7-day period. We sought to help differentiate between asymptomatic and presymptomatic positive cases.

In total, 388 individuals were tested (245 residents and 143 staff). Most residents tested were women (72%). Those testing positive had a mean age of 86.4 years (SD, 8.05). The most common comorbidities reported in the care-home residents were chronic neurological conditions and chronic heart disease. More than half of residents testing positive for SARS-CoV-2 reported having a chronic neurological condition such as dementia (55%). Most staff members in the sample were women (82%), and the mean age of staff members was 43.2 years old (SD, 18.3 years). Moreover, ~36% of the sample were smokers, none of whom tested positive for SARS-CoV-2. The most commonly reported comorbidities among staff members were chronic lung disease (9%) and diabetes mellitus (9%). Of the 245 care-home residents tested for SARS-CoV-2, 87 of 245 (36%) tested positive. Of those who tested positive, 57 of 85 (66%) were symptomatic at the time of testing, or within the 14 days prior to testing. Of 87 residents, 30 (~34%) were asymptomatic at the time of the test, or within the 14 days prior to testing. Of residents who were initially asymptomatic on testing positive for SARS-CoV-2, 12 of 30 (40%) remained asymptomatic 7 days following the test, whereas 18 of 30 (60%) developed symptoms in the week following the test. In total, 7 days after testing positive for SARS-CoV-2, 75 of 87 (86%) of residents had experienced symptoms. Of the 143 members of staff tested for SARS-CoV-2, 10 of 143 (7%) were positive. Of 10 staff members, 5 (50%) who tested positive were symptomatic at the time of the test, or in the 14 days prior to the test. These 5 were thus asymptomatic at the time of testing or within 14 days prior to the test. At the 7-day follow-up, of these 5 who were asymptomatic at the time of testing positive for SARS-CoV-2, 2 (40%) remained asymptomatic. However, 3 of these 5 staff (60%) who had been asymptomatic upon testing positive went on to develop symptoms during this time.

Overall, most of those who tested positive for SARS-CoV-2 reported symptoms between the 14 days prior to the test and the 7 days following (86%). Of these, 69% reported having at least 1 typical symptom out of cough, fever, or shortness of breath, while 13% experienced only atypical symptoms (Table 1). However, 46% of individuals experienced at least 1 atypical symptom (Table 1). Unfortunately, 27 residents (31%) who tested positive for SARS-CoV-2 died during the study period.

**Table 1.** Symptoms Reported for Residents and Staff for Those Testing Positive for SARS-CoV-2, Fr Whom Complete Information Was Obtained (n = 97)

Positive Cases	Total (n = 97), No. (%)	Residents (n = 87), No. (%)	Staff (n = 10), No. (%)	*P* Value^a^
Symptomatic when tested (or past 14 d)	62 (63.9)	57 (65.5)	5 (50)	<.001
Asymptomatic when tested (or past 14 d)	35 (36.1)	30 (34.5)	5 (50)	…
Developed symptoms at 7-day follow up	21 (21.6)	18 (20.7)	3 (30)	…
Total symptomatic at 7-d follow-up	83 (85.6)	75 (86.2)	8 (80)	<.001
Total asymptomatic at 7-d follow-up	14 (14.4)	12 (13.8)	2 (20)	…
Resident deaths	27 (27.8)	27 (31)	0 (0.0)	<.001
**Symptom details (of those symptomatic)**				
At least 1 typical symptom	57 (68.7)	49 (65.3)	8 (100)	<.001
Only atypical symptoms	11 (13.3)	11 (14.7)	0 (0)	.050
At least 1 atypical symptom	38 (45.8)	34 (45.3)	4 (50)	<.001
**Typical symptoms**				
Fever	24 (28.9)	22 (29.3)	2 (25.0)	.005
Cough	40 (48.2)	32 (42.7)	8 (100.0)	<.001
Shortness of breath/hypoxia	17 (20.5)	15 (20.0)	2 (25.0)	.026
**Atypical symptoms**				
Nasal discharge/Congestion	1 (1.2)	0 (0.0)	1 (12.5)	.632
Sneezing	1 (1.2)	0 (0.0)	1 (12.5)	.632
Sore throat	2 (2.4)	0 (0.0)	2 (25.0)	.496
Hoarseness	1 (1.2)	0 (0.0)	1 (12.5)	.632
Wheeze	0 (0.0)	0 (0.0)	0 (0.0)	…
Chest pain	2 (2.4)	1 (1.3)	1 (12.5)	.496
Acute deterioration	28 (33.7)	28 (37.3)	0 (0.0)	.004
Malaise	6 (7.2)	4 (5.3)	1 (12.5)	.269
Nausea	2 (2.4)	1 (1.3)	1 (12.5)	.535
Confusion	2 (2.4)	2 (2.7)	0 (0.0)	.535
Dizziness	0 (0.0)	0 (0.0)	0 (0.0)	…
Diarrhoea	4 (4.8)	3 (4.0)	1 (12.5)	.444
Myalgia	1 (1.2)	1 (1.3)	0 (0.0)	.632
Headache	0 (0.0)	0 (0.0)	0 (0.0)	…
Chills	1 (1.2)	0 (0.0)	1 (12.5)	.632
Anosmia	2 (2.4)	0 (0.0)	2 (25.0)	.496

a χ^2^ tests were used to compare categorical data (SPSS version 25 software).

The findings of this study suggest that individuals infected with SARS-CoV-2 may be asymptomatic at the time of infection or may present with a range of both typical and/or atypical symptoms, outside those included the current case definition for COVID-19^1-3,7^ Additionally, in this study, some individuals developed symptoms up to 1 week after they tested positive for SARS-CoV-2. During this time, individuals may have the potential to transmit the virus unknowingly to others, which may have devastating impacts in high-risk settings such as care homes. Therefore, it is critical that all residents and staff are tested in an outbreak situation to identify COVID-19 asymptomatic and presymptomatic individuals who could transmit SARS-CoV-2 before significant symptoms develop. Once it is known that someone has the infection, particularly in a care-home setting, strict infection control measures are required to contain the spread of infection. During this study, once an outbreak had been confirmed, this was managed in line with the existing public health guidance for outbreaks in a care home.

In conclusion, the findings of this study emphasize the need to identify residents and staff with atypical symptoms and to identify asymptomatic residents and staff through comprehensive and regular screening for SARS-CoV-2.
